# Crim1 has cell-autonomous and paracrine roles during embryonic heart development

**DOI:** 10.1038/srep19832

**Published:** 2016-01-29

**Authors:** Swati Iyer, Fang Yu Chou, Richard Wang, Han Sheng Chiu, Vinay K. Sundar Raju, Melissa H. Little, Walter G. Thomas, Michael Piper, David J. Pennisi

**Affiliations:** 1 School of Biomedical Sciences, The University of Queensland, Brisbane, 4072, Australia; 2 Institute for Molecular Bioscience, The University of Queensland, Brisbane, 4072, Australia; 3 Murdoch Children’s Research Institute, Melbourne, The University of Queensland, Brisbane, 4072, Australia; 4 Department of Pediatrics, University of Melbourne, The University of Queensland, Brisbane, 4072, Australia; 5 Queensland Brain Institute, The University of Queensland, Brisbane, 4072, Australia

## Abstract

The epicardium has a critical role during embryonic development, contributing epicardium-derived lineages to the heart, as well as providing regulatory and trophic signals necessary for myocardial development. Crim1 is a unique trans-membrane protein expressed by epicardial and epicardially-derived cells but its role in cardiogenesis is unknown. Using knockout mouse models, we observe that loss of Crim1 leads to congenital heart defects including epicardial defects and hypoplastic ventricular compact myocardium. Epicardium-restricted deletion of *Crim1* results in increased epithelial-to-mesenchymal transition and invasion of the myocardium *in vivo,* and an increased migration of primary epicardial cells. Furthermore, Crim1 appears to be necessary for the proliferation of epicardium-derived cells (EPDCs) and for their subsequent differentiation into cardiac fibroblasts. It is also required for normal levels of cardiomyocyte proliferation and apoptosis, consistent with a role in regulating epicardium-derived trophic factors that act on the myocardium. Mechanistically, Crim1 may also modulate key developmentally expressed growth factors such as TGFβs, as changes in the downstream effectors phospho-SMAD2 and phospho-ERK1/2 are observed in the absence of *Crim1*. Collectively, our data demonstrates that Crim1 is essential for cell-autonomous and paracrine aspects of heart development.

During development, the heart is invested with cells of the proepicardium (PE), a structure that will give rise to the epicardium, myocardial fibroblasts, cells of the coronary vasculature and some cells of the valves^1^,^2^,^3^,^4^. A critical step in the formation of these epicardium-derived lineages is the epithelial-to-mesenchymal transition (EMT) that epicardial cells must undergo to occupy the sub-epicardial space or to invade the myocardium. Some factors implicated in promoting epicardial EMT and invasion include FGFs, PDGF-A/B, TGFβ-1/2/3, and BMP-2 5,6,7,8, while these processes are restrained by NF1^9^ and WT1^10^. However, the molecular factors that control epicardial EMT and myocardial invasion, and the differentiation of epicardium-derived lineages, are incompletely understood.

Crim1 is a type-I transmembrane protein that is broadly expressed during development and which has been shown to regulate embryonic development of multiple organ systems^11^,^12^,^13^,^14^. We have previously demonstrated embryonic lethality in mice homozygous for the *Crim1*^*KST264*^ genetrap and *Crim1*^Δ*flox*^ null alleles^11^,^15^, and that homozygous *Crim1*^*KST264/KST264*^ mice display defects in organ systems including the kidney, limb, eye and placenta. These mice die perinatally on a C57BL6 background^11^. The *Crim1*^Δ*flox/*Δ*flox*^ mice display similar defects but there exist variations in severity and penetrance of the phenotypes, likely due to differences in the type of mutation used to generate each mouse line^15^. The *Crim1*^Δ*flox*^ homozygotes are also embryonic lethal on the C57BL6 background^15^. The underlying role of this molecule *in vivo* is complex, and likely involves interactions with growth factors and the extracellular milieu. Crim1 is homologous to the BMP-regulating protein Chordin, and contains six von Willebrand factor, type C-like cysteine-rich repeats (CRRs) through which it binds a broad range growth factors including TGFβ, BMP, VEGF and PDGF, when Crim1 is co-expressed in the same cell as the growth factor^12^,^13^. Moreover, Crim1 can variably have an agonistic or antagonistic effect on BMP signaling^13^,^16^,^17^, although this activity may be context-dependent. In the heart, it is expressed in PE-derived lineages, including the epicardium, and coronary vasculature^11^, however, its role in cardiogenesis, along with the growth factors it interacts with at different stages, is unclear.

In this study, we demonstrate essential autocrine and paracrine roles for Crim1 in heart development. *Crim1* loss-of-function and genetrap mice display congenital heart defects such as ventricular septal defects, epicardial defects and hypoplastic ventricular myocardium. We reveal an involvement of Crim1 in regulating epicardial cell migration into the myocardium both *in vivo* and *in vitro*. Crim1 also limits EMT in a manner independent of canonical TGFβ signaling, but possibly *via* an interaction with β-catenin, a crucial component of adherens junctions, which has been previously shown to complex indirectly with Crim1^18^. Interestingly, the cells that have lost *Crim1* and have taken up residence in the myocardium, proliferate less, and give rise to a reduced number of cardiac fibroblasts, suggesting a further cell-autonomous role for Crim1 in fate specification of cells derived from the epicardium. We also provide evidence that upon loss of *Crim1* function, compact myocardial development is aberrant, likely due to epicardial loss of *Crim1*, indicative of a paracrine role. Collectively, these findings illustrate how Crim1 and other factors function in tandem to regulate heart development.

## Results

### Expression of Crim1 during heart development using a genetrap reporter

**Figure 1:**
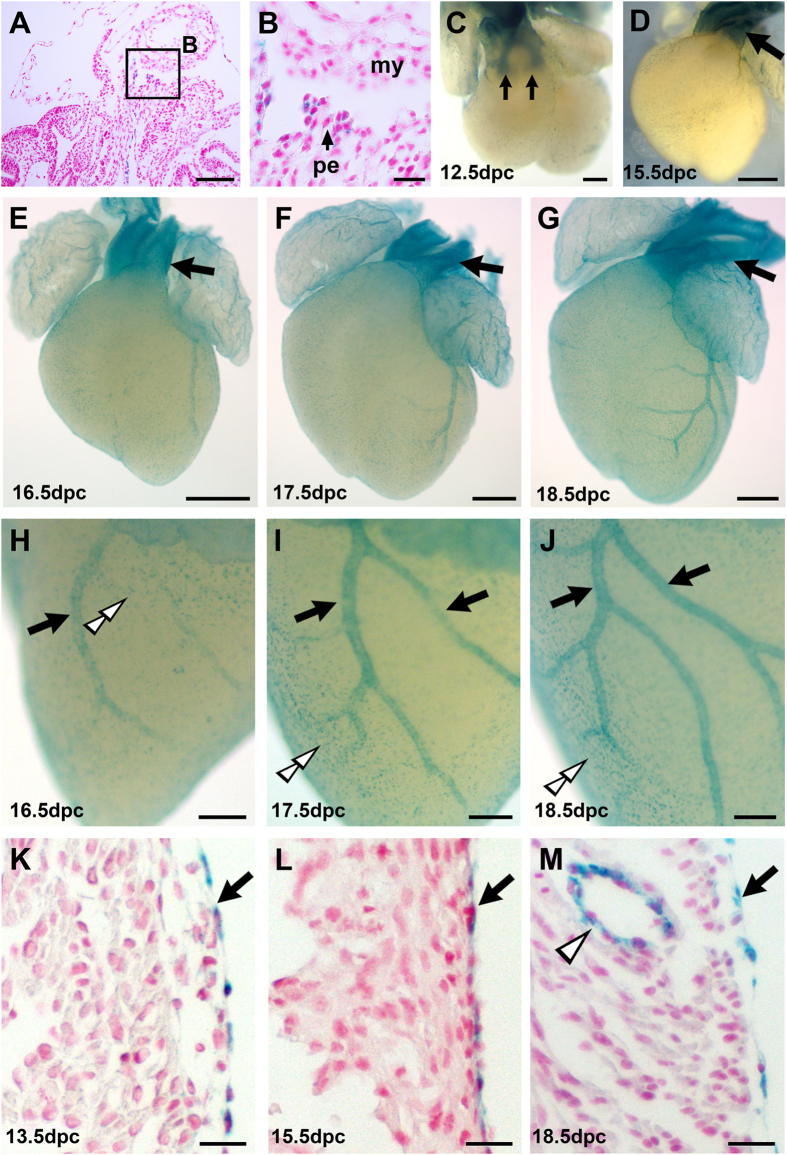
Crim1 expression as monitored by the *Crim1*^*KST264*^ genetrap reporter reveals a dynamic pattern during heart development. (**A**) micrograph of a histological section of a 9.5 dpc *Crim1*^+*/KST264*^ embryo that has been X-Gal-stained, paraffin-embedded, and counter-stained with nuclear fast red. (**B**) higher magnification view of the boxed area in (**A**). The proepicardial cells are X-Gal-positive (arrow, **B**). Note that X-Gal staining is localised close to nuclei due to the targeting of the β-geo to cell bodies that was typical of the secretory trap vector used in the genetrap screen that generated the *Crim1*^*KST264*^ allele [49]. (**C–G**) Whole-mount views of *Crim1*^+*/KST264*^hearts after X-Gal staining (blue) at 12.5 dpc (**C**), 15.5 dpc (**D**), 16.5 dpc (**E**), 17.5 dpc (**F**), and 18.5 dpc (**G**). In addition to expression in the epicardium at these stages, note the Crim1-LacZ expression-in the OFT mesenchyme (arrows) at 12.5 dpc, and the smooth muscle of the great vessels (arrows, **D–G**). (**H–J**) Magnified left lateral views of the ventricles of the hearts shown in (**E**–**G**) respectively. Note the Crim1-LacZ expression in the developing coronary vasculature (arrows) and in the epicardium (double open arrowheads). (**K–M**) histological sections Crim1-LacZ expression in the epicardium (arrows) and (**M**) in the coronary vasculature (open arrowhead). my, myocardium; pe, proepicardium. Scale bar (**A**) 100 μm; (**B**) 20 μm; (**C**) 200 μm; (**D**–**G**) 500 μm; (**H**–**J**) 200 μm; (**K**–**M**) 20 μm.

To investigate Crim1 expression in the developing heart, we used a genetrap line for *Crim1*, *Crim1*^*KST264*^ 11 that allowed the use of a *LacZ* reporter under the control of the *Crim1* locus. We observed *Crim1* expression in the proepicardium at 9.5 days post coitum (dpc), and in the epicardium at all stages during cardiac development (Fig. 1A–M). *Crim1* was also expressed in coronary vascular smooth muscle and coronary endothelial cells, as reported previously^11^. Crim1 expression in the endocardium and valve leaflets was confirmed by performing X-Gal staining coupled with immunohistochemical analyses against periostin, a mesenchymal cell marker (Supplementary Fig. 1A–E), and Crim1 expression in coronary vascular smooth muscle cells was confirmed using transgelin double labelling (Supplementary Fig. 1F). The dynamic expression pattern observed suggests that *Crim1* plays a role in heart development.

### Developmental defects in hearts of *Crim1* mutant embryos

**Figure 2:**
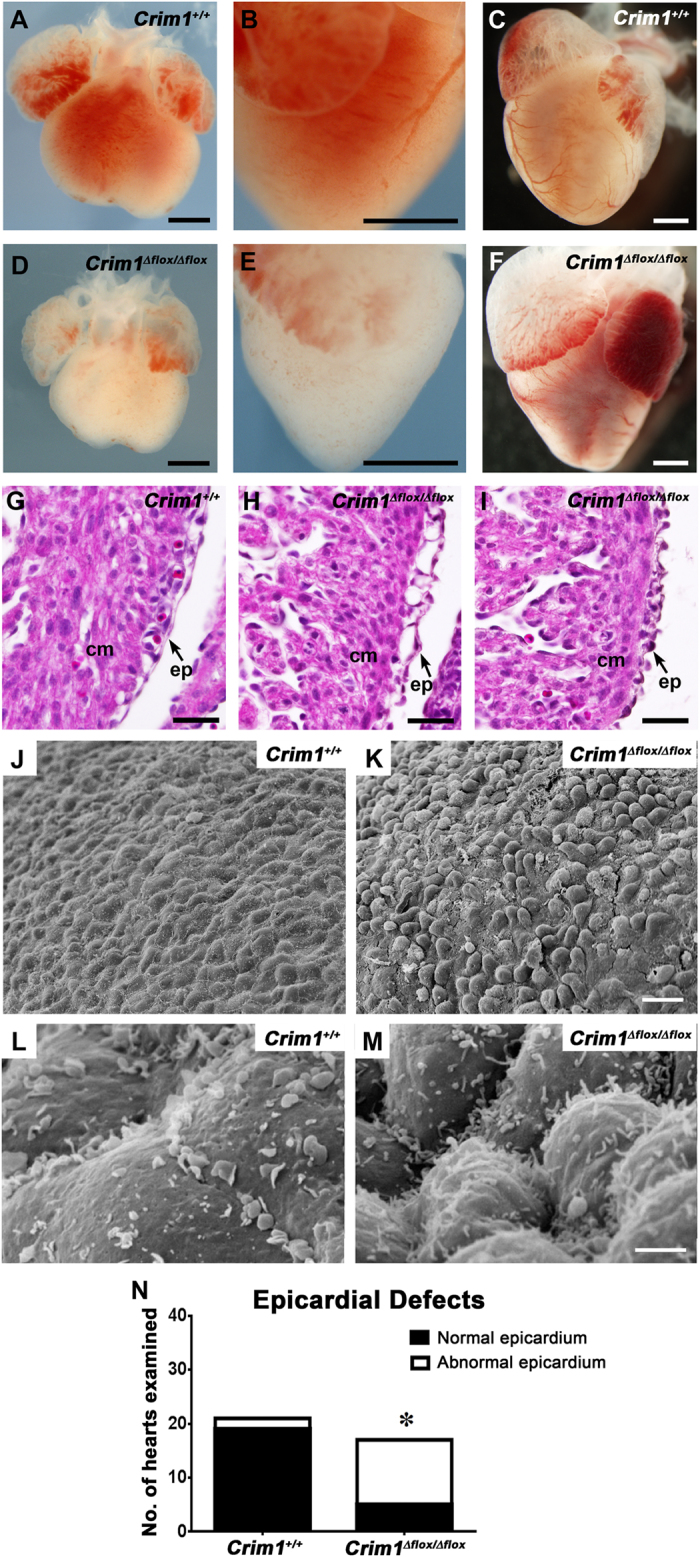
Developmental defects in hearts of *Crim1*^Δ*flox/*Δ*flox*^ embryos. (**A,B**,**D,E**) Representative whole-mount views of 14.5 dpc embryonic hearts (**A,B**, *Crim1*^+/+^; **D,E** *Crim1*^Δ*flox/*Δ*flox*^). (**B**,**E**) Magnified, left lateral views of (**A**,**D**) respectively. (**C**,**F**) Representative whole-mount views of 17 dpc embryonic hearts (**C**, *Crim1*^+*/*+^; (**F**) *Crim1*^Δ*flox/*Δ*flox*^). Note the reduced size of the ventricles in the *Crim1*^Δ*flox/*Δ*flox*^ heart (**D**–**F**). Epicardial defects in *Crim1*^Δ*flox/*Δ*flox*^ hearts. (**G**–**I**) Hematoxylin and eosin-stained sections of the ventricular region of 13.5 dpc (**G**) *Crim1*^+*/*+^ and (**H**,**I**) two different *Crim1*^Δ*flox/*Δ*flox*^ hearts. Note the irregular appearance of the epicardium in *Crim1*^Δ*flox/*Δ*flox*^ embryos, with one example showing blebbing (**H**). (**J–M**) Scanning electron micrographs of the ventricular surface of 13.5 dpc *Crim1*^+*/*+^ (**J,L**) and *Crim1*^Δ*flox/*Δ*flox*^ (**K,M**) hearts. (**K**) Note the loss of the even spacing of cells of the mesothelial epicardium, and (**M**) diffusely arranged microvilli. (**N**) Quantification of penetrance of epicardial defects, specifically either epicardial blebbing or a loss of squamous morphology, between *Crim1*^+*/*+^ and *Crim1*^Δ*flox/*Δ*flox*^ hearts at 13.5 dpc. Z-score -3.8801. *P < 0.0001. cm, compact myocardium; ep, epicardium. Scale bars; A–F, 500 μm; G-I, 50 μm; J-K, 20 μm; L-M, 2 μm.

The expression pattern of *Crim1* led us to investigate whether there were heart defects exhibited by *Crim1* mutant mice. Examination of hearts from embryos homozygous for the *Crim1*^*KST264*^ genetrap mutant (Supplementary Fig. 2A–L) revealed epicardial malformations and blebbing (Supplementary Fig. 2J–L), as well as a reduced ventricular size (Supplementary Fig. 2A–C) and muscular ventricular septal defects (Supplementary Fig. 2D–I). Similarly, *Crim1*^Δ*flox/*Δ*flox*^ embryonic hearts had reduced ventricle size at 14.5 dpc (Fig. 2A,B,D,E), and distended atria and reduced ventricular size at 17.0 dpc (Fig. 2C,F). Histological analysis of *Crim1*^Δ*flox/*Δ*flox*^ hearts at 13.5 dpc revealed abnormal epicardial morphology, including epicardial blistering (Fig. 2G–I,N), findings supported by scanning electron microscopic analysis of *Crim1*^Δ*flox/*Δ*flox*^ hearts, which revealed a loss of the regular, cobblestone appearance of the epicardium (Fig. 2J–M).

These phenotypes may have arisen from deficits in early PE formation. To address this, we used *in situ* hybridization on *Crim1*^Δ*flox/*Δ*flox*^ embryos for the PE markers *Gata4* and *Tbx18*. We found that PE formation and morphology, and PE marker expression, was similar between *Crim1*^Δ*flox/*Δ*flox*^ and control embryos (Supplementary Fig. 3), indicating that Crim1 is not necessary for initial outgrowth of the PE. From this we infer that Crim1 may regulate adhesion or migration at a later time point within the developing epicardium.

### Crim1 is necessary for normal compact myocardial development

**Figure 3:**
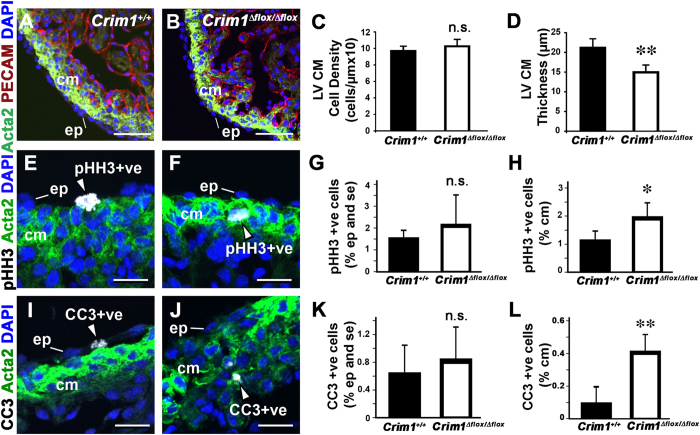
Crim1 is necessary for normal compact myocardial development. (**A**,**B**) Merged confocal images of left ventricular sections of 13.0 dpc hearts from *Crim1*^+*/*+^ (**A**) and *Crim1*^Δ*flox/*Δ*flox*^ (**B**) hearts stained with DAPI (blue), PECAM-1 (red), and actin (green). (**C**) Compact myocardium cell density was unchanged in *Crim1*^Δ*flox/*Δ*flox*^ hearts. (**D**) Reduced ventricular compact myocardial thickness in *Crim1*^Δ*flox/*Δ*flox*^ hearts at 13.0 dpc (n = 6–8). (**E,F**) Merged confocal images of ventricular sections of 13.0 dpc hearts stained with phospho- Histone H3 (pHH3, white), DAPI (blue), and actin (green). An epicardial cell (arrowhead, **E**) and myocardial cell (arrowhead, **F**) immuno-positive for pHH3 are shown. (**G**) No difference in proliferation in the epicardium of *Crim1*^Δ*flox/*Δ*flox*^ hearts. (**H**) An almost two-fold increase in proliferation in the compact myocardium of *Crim1*^Δ*flox/*Δ*flox*^ hearts. (**I**,**J**) Examples of merged confocal images of ventricular sections of 13.0 dpc hearts stained with cleaved caspase 3 (CC3, white), DAPI (blue), and actin (green). An epicardial cell (arrowhead, **I**) and myocardial cell (arrowhead, **J**) immuno-positive for CC3 are shown. (**K**) No difference in apoptosis in the epicardium of *Crim1*^Δ*flox/*Δ*flox*^ hearts. (**H**) A four-fold increase in apoptosis in the compact myocardium of *Crim1*^Δ*flox/*Δ*flox*^ hearts. cm, compact myocardium; ep, epicardium; n.s., not significant. (n = 4–5), *P < 0.05; **P < 0.01. Scale bars (**A**,**B**) 50 μm; (**E**,**F**,**I**,**J**) 20 μm.

As we had observed a reduction in ventricular size at 14.5 dpc in *Crim1*^Δ*flox/*Δ*flox*^ hearts, we investigated whether there were any changes in thickness of the compact myocardium at 13.0 dpc (when the myocardium has started to develop) that could underlie this phenotype. We observed a reduced compact myocardial thickness in *Crim1*^Δ*flox/*Δ*flox*^ hearts at 13.0 dpc, a finding that was not due to changes in cell density (Fig. 3A–D). Interestingly, this phenotype is shared by other mutants that lack epicardium or epicardium-derived factors^19^. To further examine the nature of this defect, we analyzed the proliferation marker, phospho-histone H3 (pHH3; Fig. 3E–F), and the apoptotic marker, cleaved caspase 3 (CC3; Fig. 3I–J). We found an almost two-fold increase in proliferation, and a four-fold increase in apoptosis in the compact myocardium of *Crim1*^Δ*flox/*Δ*flox*^ hearts (Fig. 3H,L). No change was observed in proliferation or apoptosis in epicardial or sub-epicardial cells of *Crim1*^Δ*flox/*Δ*flox*^ hearts at this age (Fig. 3G,K). This suggests that the loss of Crim1 culminates in elevated cell death within the compact myocardium during development, but whether it is an autocrine effect of Crim1 expressed within the compact myocardium, or epicardially-expressed Crim1 acting in a paracrine manner, is unknown.

**Figure 4:**
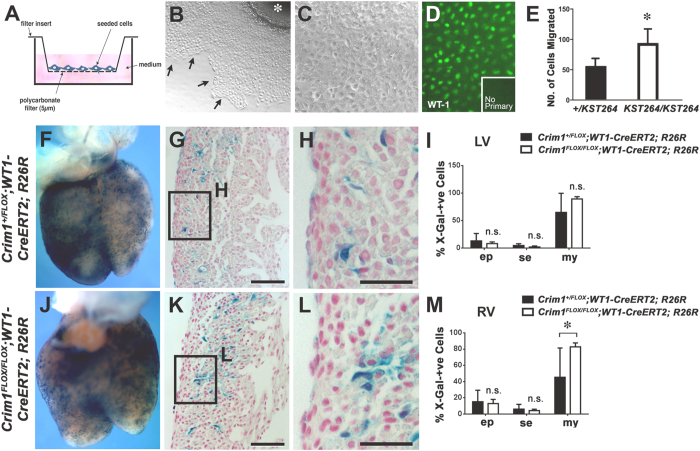
A cell-autonomous requirement for Crim1 to control epicardial migration and myocardial invasion *in vitro* and *in vivo*. (**A**) Schematic of the transwell assay used for the *in vitro* migration assay. (**B**) Representative micrograph of a primary 11.5 dpc ventricular explant after attachment and migration of epicardial cells. The front of the epicardial monolayer is indicated (arrows). The primary explant is denoted by an asterisk. (**C**) Primary epicardial cells after removal of the ventricular explant, passage and subsequent culture. Note maintenance of a “cobblestone” appearance of the cellular monolayer. (**D**) Epicardial identity after enrichment was confirmed by immunostaining for WT1 (green nuclear signal). The no-primary antibody control showed no signal (inset). (**E**) *Crim1*^*KST264/KST264*^ primary epicardial cells showed increased migration *in vitro* relative to controls (n = 3–5 and representative of three independent experiments; *P < 0.05). (**F**,**J**) Whole-mount dorsal views of 15.5 dpc embryonic hearts after X-Gal staining (blue) of *Crim1*^+*/FLOX*^*; WT1-CreERT2; R26R* and *Crim1*^*FLOX/FLOX*^*; WT1-CreERT2; R26R* genotype, respectively. Two doses of Tamoxifen were administered at 9.5 dpc and 10.5 dpc prior to harvesting the embryos. (**G**,**K**) Histological sections of hearts from *Crim1*^+*/FLOX*^*; WT1-CreERT2; R26R* and *Crim1*^*FLOX/FLOX*^*; WT1-CreERT2; R26R* 15.5 dpc embryos, respectively, and (**H**,**L**) magnified views of boxed regions showing X-Gal-positive epicardial, sub-epicardial and intramural cells. (**I,M**) Quantification of X-Gal-positive cells showing increased myocardial EPDCs in the right ventricles of mutant hearts (n = 4–6; *P < 0.05). ep, epicardial; se, sub-epicardial; my, intramyocardial; LV, left ventricle; RV, right ventricle. Scale bars, (**G**,**K**) 20 μm (**H**,**L**) 10 μm.

To address this question, we analyzed the potential autocrine role of Crim1 in compact myocardial development by deleting *Crim1* from the cardiomyocytes in the myocardium of the developing heart using the *Mlc2v-Cre* line. This line is efficient and restricted to ventricular cardiomyocytes^20^,^21^,^22^,^23^. Cardiomyocyte proliferation and survival upon myocardial loss of *Crim1* in the ventricular myocardium were examined using pHH3 and CC3 immunocytochemistry, respectively. Quantification of cells expressing both X-Gal and pHH3, or cells expressing X-Gal and CC3 in the compact myocardium at 13.5 dpc revealed no significant differences in the number of either pHH3- and CC3-positive cells between control and mutant hearts (Supplementary Fig. 4). Moreover, no significant change in compact myocardium thickness was observed in these mice (Supplementary Fig. 4). From this, and the fact that the compact myocardium phenotype is observed before the epicardial cells have undergone EMT and invaded the compact myocardium, we infer that the myocardial deletion of *Crim1* does not give rise to deficits in the compact myocardium, but rather that epicardial Crim1 regulates the development of the myocardium in a paracrine fashion. In order to substantiate this, we crossed the conditional *Crim1*^*FLOX*^ line to a strain that would enable inducible ablation of *Crim1* from the epicardium (*WT1-CreERT2*). The *WT1-CreERT2* line has previously been shown to be specific to the epicardium and epicardium-derived lineages^24^. We further corroborated this by immunohistochemical analysis using the cardiomyocyte marker MF20, which revealed limited overlap between the X-Gal staining and MF20 immunoreactivity (Supplementary Fig. 5D,E). Two doses of Tamoxifen were administered at 9.5 dpc and 10.5 dpc prior to harvesting the embryos, and epicardial-restricted Cre activity as assessed by the R26R LacZ reporter was confirmed in embryonic hearts from 12.5 dpc (Fig. 4F,G,J,K, and data not shown). Unlike the hearts of *Crim1*^Δ*flox/*Δ*flox*^ mice, the phenotype of reduced myocardial wall thickness was not recapitulated in the *WT1-CreERT2* hearts at 15.5 dpc (data not shown). This could be due to incomplete penetrance and variable expressivity; a common occurrence with Cre lines, including the *WT1-CreERT2* line^24^. Indeed, upon quantification of the percentage of epicardial cells that had lost *Crim1* (pooled genotypes, n = 6; data not shown), we saw that in the left and right ventricles, there were still 62.5% of epicardial cells in which *Crim1* was not ablated, which could compensate for any myocardial defects that may have arisen with a complete lack of *Crim1*.

### Cell-autonomous requirement for Crim1 to control epicardial migration and myocardial invasion *in vitro* and *in vivo*

Given the potential role for epicardially-derived Crim1 in regulating myocardial development, as well as the epicardial defects observed in the hearts of conditional null and genetrap mutant embryos, we next examined the role of Crim1 in epicardial development. We first sought to determine whether *Crim1* mutant epicardial cells exhibited migration defects. To do this we cultured primary embryonic epicardial cells from the *Crim1*^*KST264*^ genetrap line in a transwell migration assay (modified Boyden chamber assay). The *Crim1*^*KST264*^ allele in these experiments allowed us to monitor the continued expression of Crim1 by X-Gal staining in representative examples of explant cultures. In all cases, ubiquitous *Crim1-LacZ* expression was observed in primary epicardial cells (data not shown). We found that primary epicardial cultures from *Crim1*^*KST264/KST264*^ hearts, immunostained with WT1 as an epicardial marker to confirm epicardial identity after enrichment (Fig. 4D), exhibited an increased rate of migration relative to heterozygote controls (Fig. 4A–E), suggesting that the loss of Crim1 from the developing epicardium resulted in increased epicardial migration.

To confirm these observations *in vivo*, and to also investigate changes in EMT, we used the *WT1-CreERT2* line and assessed the location of X-Gal-positive cells at 15.5 dpc in *Crim1*^*FLOX/FLOX*^*; WT1-CreERT2; R26R* and littermate heterozygous controls, determining whether labelled cells remained epicardial, or had undergone EMT and migrated to the sub-epicardium or to an intramural myocardial location. We found that loss of *Crim1* in epicardial cells resulted in an increase in the number of epicardial cells that had undergone EMT and invaded the myocardium of the right ventricle relative to controls (Fig. 4F–M). This finding was confirmed using a second Cre driver, namely *WT1-Cre*^25^ (data not shown). These *in vivo* data indicate that the loss of *Crim1* in the epicardium results in increased epicardial EMT, and are consistent with the primary epicardial culture migration experiments indicating that the loss of Crim1 function results in increased epicardial cell migration.

**Figure 5:**
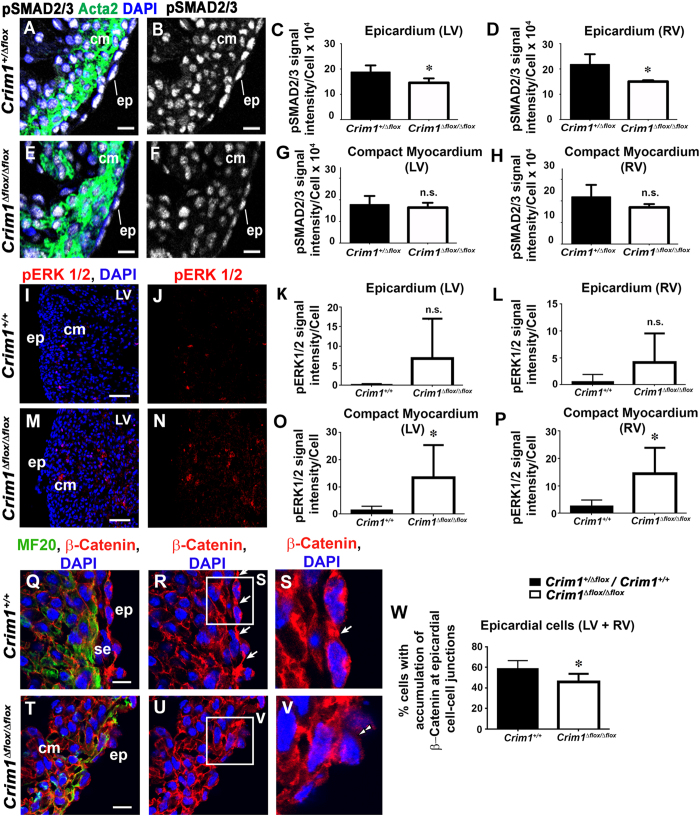
Changes in epicardial and myocardial signalling, alongside localisation of β-catenin in *Crim1*^Δ*flox/*Δ*flox*^ hearts. (**A,B**,**E,F**), Confocal images of ventricular sections of 13.0 dpc hearts from *Crim1*^+*/*Δ*flox*^ (**A,B**) and *Crim1*^Δ*flox/*Δ*flox*^ (**E,F**) embryos stained for phospho-SMAD2/3 (white). (**A,B**) merged images of DAPI (blue) and actin (green) to delineate the myocardium with the phospho-SMAD2/3 (white) from B and F, respectively. (**C,D,G,H**) There was a reduction in phospho-SMAD2 signal intensity in epicardial cells of left and right ventricles of *Crim1*^Δ*flox/*Δ*flox*^ hearts, but the levels in compact myocardial cells was unchanged (n = 4–5). Confocal images of ventricular sections of 13.5 dpc hearts from *Crim1*^+*/*+^ (**I,J**) and *Crim1*^Δ*flox/*Δ*flox*^ (**M,N**) embryos stained for phospho-ERK1/2 (red). (**I,M**) merged images of DAPI (blue) and phospho-ERK1/2 (red). (**K,L,O**,**P**), There was an increase in phospho-ERK1/2 signal intensity in compact myocardial cells of left and right ventricles of *Crim1*^Δ*flox/*Δ*flox*^ hearts, but the levels in epicardial cells was unchanged (n = 8–6). (**Q,R,T,U**) Confocal images of ventricular sections of 13.5 dpc hearts from *Crim1*^+*/*+^ (**Q,R,S**) and *Crim1*^Δ*flox/*Δ*flox*^ (**T,U,V**) embryos stained for β-catenin (red). (**Q,T**) merged images of DAPI (blue) and MF20 (green) to delineate the epicardium with the β-catenin (red) from (**R,S**,**U,V**) respectively. (**S,V**) magnified views of boxed regions in (**R**,**U**). Note β-catenin distribution at epicardial cell-cell junctions of *Crim1*^+/+^ hearts (arrows, **R,S**), and a decreased accumulation at epicardial cell junctions in mutants (double arrowheads, **V**). (**W**) the percentage of epicardial cells with an accumulation of β-catenin at cell junctions (n = 5–6). cm, compact myocardium; ep, epicardium; s.ep, sub epicardium. n.s., not significant. *P < 0.05. Scale bars, (**A,B**,**E,F**) 10 μm (**I,J**,**M,N**) 50 μm (**Q,R**,**T,U**) 10 μm.

To gain insight into the mechanism by which Crim1 may regulate EMT and the migration of epicardial cells, we next investigated whether growth factor signalling in both the epicardium and the myocardium is perturbed in *Crim1*-deficient mice. Numerous growth factors have been implicated in epicardial EMT, including TGFβ and BMPs (acting via TGFβ receptors)^5^,^6^,^7^,^8^. As Crim1 has been shown to bind such growth factors in cell culture binding experiments^12^,^13^,^26^, and potentially regulate their activity *in vivo*^12^,^26^, we examined whether there were changes in canonical TGFβ or BMP signalling at the time of epicardial EMT and invasion, using the *Crim1*^Δ*flox*^ line. To do this we performed indirect immunofluorescence for phospho-SMAD2 and phospho-SMAD1/5 as proxies to assess canonical TGFβ and BMP signalling, respectively. We also investigated whether there were changes in downstream effectors such as ERK1/2 and AKT as further read-outs of aberrant signal transduction. There was a reduced level of phospho-SMAD2 in the epicardium of *Crim1*^Δ*flox/*Δ*flox*^ hearts at 13.0 dpc (77% and 69% of control values for the left and right ventricular epicardium, respectively) (Fig. 5A–F). Despite the increased epicardial EMT and invasion suggested by the epicardial-restricted loss of *Crim1* function, these results suggest that canonical TGFβ signalling was reduced in the epicardium of *Crim1*^Δ*flox/*Δ*flox*^ hearts. There was no change in levels of phospho-SMAD1/5 (Supplementary Fig. 6A–H), phospho-AKT (Supplementary Fig. 6I–P) and phospho-ERK1/2 (Fig. 5I–N) in the epicardium of *Crim1*^Δ*flox/*Δ*flox*^ hearts relative to wildtype controls. At 13.0 dpc, *Crim1*^Δ*flox/*Δ*flox*^ hearts showed comparable levels of ventricular compact myocardial phospho-SMAD2 (Fig. 5A,B,E–H), phospho-SMAD1/5 (Supplementary Fig. 6A–H) and phospho-AKT (Supplementary Fig. 6I–P) relative to controls. However, we observed that there was an increase in both the number of phospho-ERK1/2 positive cells (for example, 29% of cells were phospho-Erk1/2-positive in the left ventricle of the mutant, compared to 6% in the left ventricle of the control; P < 0.05, *t*-test), as well as an increase in the phospho-ERK1/2 signal intensity per cell in the ventricular compact myocardium of mutant hearts relative to controls (Fig. 5I,J,M–P). There was also no significant difference in the distance that pERK1/2 positive cells were from the epicardium in both control and mutant hearts (data not shown). Together with the lack of compact myocardial changes observed in the myocardium-restricted *Crim1* knock-out, this indicates that Crim1 could play a paracrine role in regulating myocardial development, likely through the regulation of epicardium-derived factors.

The adhesion molecule β-catenin is an important regulator of epithelial stability and EMT, and Crim1 has been shown to interact indirectly with β-catenin^18^. To investigate the role of Crim1 in altering EMT and migration, we analyzed the localisation of β-catenin^27^ in *Crim1*^+*/*+^ and *Crim1*^Δ*flox/*Δ*flox*^ hearts at 13.5 dpc. We found that there was a decrease in the percentage of epicardial cells that displayed an accumulation of β-catenin at epicardial cell-cell junctions in mutant hearts (Fig. 5Q–W). When considered in light of the findings that increased EMT and myocardial invasion occurs in the hearts of *Crim1* mutant mice, this finding suggests that Crim1 normally regulates the stability of intercellular junctions within the epicardium. Analysis of filamentous actin to assess the cytoskeletal remodelling that accompanies EMT and migration revealed no visible changes in stress fibre morphology between mutant and wildtype hearts at 13.5 dpc (Supplementary Fig. 7). The data reveal a cell-autonomous role for Crim1 in controlling epicardial migration, EMT and invasion in primary cell culture and *in vivo*, which appears to rely on interactions with β-catenin.

### Cell-autonomous requirement for Crim1 to control proliferation and fate specification of EPDCs

**Figure 6:**
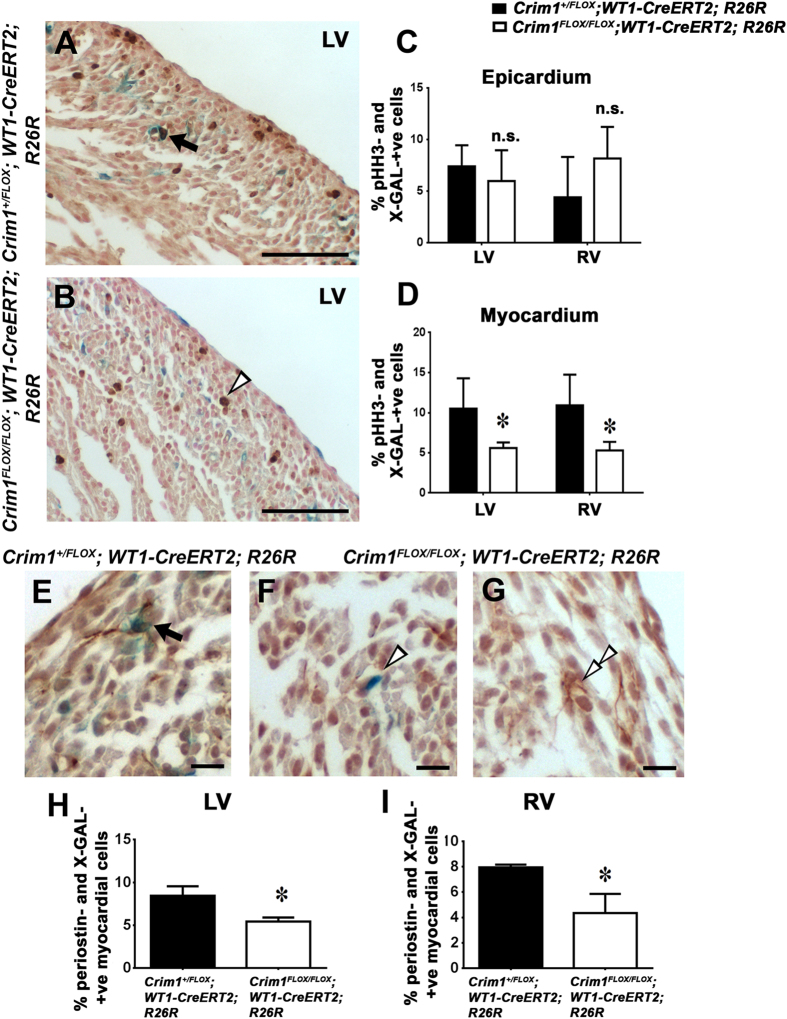
Epicardium-restricted Crim1 loss-of-function results in decreased proliferation of EPDCs and cardiac fibroblasts in both left and right ventricles. (**A,B**) phospho-Histone H3 immunohistochemistry on X-Gal stained *Crim1*^+*/FLOX*^; +*/WT1-CreERT2; R26R* and *Crim1*^*FLOX/FLOX*^; +*/WT1-CreERT2; R26R* sections at 15.5 dpc, counter stained with 1% DAB followed by NFR staining for visualisation of nuclei, showing (**A**) pHH3- and X-Gal-positive nuclei (arrow) and (**B**), a pHH3-positive nucleus (open arrowhead). (**C,D**) Quantification of proliferating EPDCs showing no change in the epicardium, but a significant reduction in the number of pHH3-positive EPDCs in mutant hearts compared to control in the myocardium (n = 6–4). (**E,G**) Periostin immunohistochemistry on X-Gal stained *Crim1*^+*/FLOX*^; +*/WT1-CreERT2; R26R* and *Crim1*^*FLOX/FLOX*^; +*/WT1-CreERT2; R26R* sections at 17.5 dpc, counter stained with 1%DAB followed by Hematoxylin staining, showing (**E**), a periostin- and X-Gal-positive cardiac fibroblast (arrow), (**F**), an X-Gal-positive EPDC (open arrowhead) and (**G**) a periostin-positive cardiac fibroblast (double open arrowheads). (**H,I**) quantification of % periostin-positive cells among X-Gal-positive myocardial cells in left and right ventricles, showing a decrease in number of cardiac fibroblasts (n = 3–5). *P < 0.05. n.s., not significant; LV, left ventricle; RV, right ventricle. Scale bars (**A**,**B)** 50 μm; (**E**–**G**) 10 μm.

The increase in the number of EPDCs that had migrated into the myocardium prompted us to further investigate the proliferative ability and differentiation potential of these EPDCs. We used the *WT1-CreERT2* and *Crim1*^*FLOX*^; *R26R* lines and assessed EPDC proliferation at 15.5 dpc using pHH3 (Fig. 6A,B). Quantification of X-Gal expressing cells that were pHH3-positive in the myocardium of left and right ventricles revealed a significant reduction in proliferation of intramyocardial cells in *Crim1*^*FLOX/FLOX*^; +/*WT1-CreERT2; R26R* hearts (Fig. 6D). Quantification of pHH3-positive cells among X-Gal-positive epicardial cells, however, revealed comparable results (Fig. 6C). This demonstrates that deleting *Crim1* from the epicardium can perturb proliferation of epicardium-derived cells, even though there is increased migration into the myocardium.

To address whether changes in epicardial cell fate occurred due to loss of Crim1, we performed qPCR analyses on ventricular tissue from 17.5 dpc *Crim1*^Δ*flox/*Δ*flox*^ and *Crim1*^+*/*+^ hearts using vascular smooth muscle, fibroblast and cardiomyocyte markers. No significant changes in these markers were observed, although there was a trend for a reduction in the fibroblast marker *Collagen1a* (P = 0.0556; Supplementary Fig. 8A). To specifically examine the effect of epicardial *Crim1* deletion on the differentiation of EPDCs into cardiac fibroblasts, we used the *WT1-CreERT2* line to perform IHC using periostin, another fibroblast marker, on heterozygote control and mutant *WT1-CreERT2* heart sections at 17.5 dpc. Quantification of the proportion of periostin- and X-Gal-positive cells in the myocardium revealed a significant decrease in the expression of periostin in the left and right ventricles of the mutant (Fig. 6E–I). This suggests that the number of cardiac fibroblasts were reduced, and the secretory phenotype of cardiac fibroblasts was perturbed upon epicardial *Crim1* loss-of-function, indicating a possible cell-autonomous requirement for Crim1 to regulate fate specification of EPDCs into fibroblasts.

## Discussion

The epicardium gives rise to crucial components of the developing heart, and while the differentiation of EPDCs into cardiomyocytes and coronary endothelial cells remains a controversial topic, reactivated epicardial cells after myocardial damage have been shown to give rise to fibroblast and smooth muscle cells^28^,^29^,^30^,^31^,^32^. The epicardium also has a critical role in providing instructive cues and trophic factors for the developing myocardium. Although certain epicardium-derived factors are believed to act on the myocardium in a paracrine manner, the nature of this interaction is not entirely understood. Here, we demonstrate an essential role for Crim1 in the epicardium during heart development. Crim1 appears to regulate epicardial EMT and invasion, fate specification of EPDCs into cardiac fibroblasts, and modulation of growth factor activity. As a result, we infer that Crim1 controls compact myocardial development via regulation of epicardium-derived paracrine factors.

Crim1 is expressed in a large number of tissues in a spatially and temporally regulated manner and has been shown to have diverse extracellular and intracellular functions^11^,^12^,^13^,^14^,^18^,^33^,^34^. This study focused determining the role of Crim1 in the cardiogenesis by using different transgenic mouse lines. We have observed that *Crim1* expression in the heart begins in the proepicardium, although it is not necessary for PE specification, and continues in the epicardium and epicardium-derived lineages. *Crim1*^*KST264/KST264*^ homozygotes showed a disrupted epicardial layer, reduced ventricular size, and prominent ventricular septal defects. Hearts of both *Crim1*^Δ*flox/*Δ*flox*^ and *Crim1*^*KST264/KST264*^ homozygotes at 13.5 dpc showed irregular epicardial morphology indicative of EMT and adhesion defects. Indeed, epicardial deletion of Crim1 resulted in an increase in epicardial cell EMT and myocardial invasion. Similarly, primary epicardial cultures showed increased migration upon loss of Crim1. However, epicardial cells lacking Crim1 also showed reduced proliferation and an overall reduction in cardiac fibroblast marker expression. Overall this suggests that loss of Crim1 in epicardial cells affects cell phenotype, proliferation and fate.

Growth factors like BMPs and TGFβs that can be bound by Crim1 promote epicardial EMT and invasion^5^,^7^,^8^. Canonical BMP signalling via the SMAD1/5 pathway, AKT signalling and ERK1/2 in the epicardium appear to be largely independent of Crim1 in *Crim1*^Δ*flox/*Δ*flox*^ hearts. Indeed, we observed a paradoxical reduction in epicardial TGFβ signalling in *Crim1*^Δ*flox/*Δ*flox*^ embryos. This suggests that, in the epicardium, Crim1 may play a role other than growth factor regulation with Crim1 mutants representing an uncoupling of these growth factor signalling pathways and epicardial EMT. β-catenin distribution, however, at epicardial cell-cell junctions was altered in the absence of Crim1. Interestingly, *Xenopus* Crim1 has been shown to interact via its cytoplasmic domain with β-catenin and N-cadherin, identifying a role for Crim1 in the formation or stabilization of cadherin-dependent junctional complexes in epithelial cells^18^. Though Crim1 was not always present at sites of cadherin expression, it may interact with cadherins and β-catenin within the endoplasmic reticulum, where complex formation between the 3 interacting partners would occur, possibly displacing an existing stabilization mechanism^18^. Crim1-mediated sequestration of β-catenin from junctional complexes could also limit Wnt signalling. Hence, the link between Crim1 and the β-catenin pathway, particularly in the developmental interactions between the epicardium and myocardium, remains to be explored.

The altered myocardial thickness in the absence of Crim1 suggests a role for Crim1 in the regulation of signalling molecules from the epicardium, and possibly the myocardium as well, crucial for normal myocardial development. The underlying mechanism that controls the developing myocardium is not clear. Mouse mutants, including knockouts of VCAM-1, α4 integrin and WT-1, and mouse lines with altered retinoic acid signalling, show perturbation of epicardial formation with secondary myocardial defects during development^35^,^36^,^37^,^38^. Furthermore, microsurgical inhibition of epicardium formation in avian embryos results in hypoplastic ventricular compact myocardium^39^,^40^. Similarly, we observed a reduced compact myocardial thickness in *Crim1*^Δ*flox/*Δ*flox*^ hearts at 13.0 dpc. However, epicardium-specific deletion of *Crim1* did not recapitulate this phenotype, possibly due to incomplete penetrance within the *WT1-CreERT2* line during embryonic stages. Similarly, the myocardial depletion of *Crim1* did not culminate in a thinner myocardium, nor did it reveal an effect on cardiomyocyte proliferation or apoptosis. In light of the fact that reciprocal signalling between the epicardium and myocardium is required for proper myocardial development to ensue, it is plausible that both epicardial and myocardial Crim1 are required for normal formation of the myocardium. An alternative interpretation of these results is that there is a non-cell-autonomous requirement for epicardial Crim1 in myocardial development. Embryonic cardiomyocytes respond to a variety of signalling molecules and express a broad range of receptors which can bind mitogenic factors^19^. Indeed, IGFs, PDGFs and VEGFs secreted by the epicardium may act as epicardial mitogens that stimulate ventricular development and cardiomyocyte proliferation^41^,^42^. IGFBPs are known to regulate the activity of IGFs^43^. The IGFBP domain of Crim1 has been shown to possess sequence similarity to IGFBP-7^14^ potentially allowing it to bind IGFs and insulin to mediate the regulation of their downstream signalling. This is in accordance with our observation that ERK1/2 signalling in the myocardium of *Crim1*^Δ*flox/*Δ*flox*^ hearts is increased. Crim1 may serve to sequester growth factors secreted by the epicardium, without necessarily affecting the range of growth factor action in *Crim1*^Δ*flox/*Δ*flox*^ hearts, where these may be free to signal to the adjacent myocardium. This has been reported previously with respect to a role for Crim1 in VEGF sequestration in the glomerulus^12^. Crim1 and the growth factors it binds have been reported to be co-expressed in the same cell^12^,^13^, which would be consistent with a role in tethering epicardially-derived growth factors. Prolonged ERK1/2 phosphorylation has been shown to lead to cell death, whereas transient activation drives proliferation^44^. Thus, an increase in phospho-ERK1/2 activity in the compact myocardium could have a pro- or anti-apoptotic effect, depending on the activation of downstream effectors. Crim1 has also been shown to reduce the production and secretion of BMPs^13^. A lack of Crim1 could lead to increased growth factor production and signalling, and result in a high cell turnover in the myocardium of mutant hearts as a result of co-activation of proliferation and apoptosis. It is important to note that the antagonistic or agonistic functions of Crim1 are context-dependent and could change for different cell types within the heart and at different stages of development.

We also observed a decrease in the proliferative ability of EPDCs in the myocardium of *Crim1*^*FLOX/FLOX*^*; WT1-CreERT2; R26R* hearts, in spite of an increased migration of cells derived from the epicardium as seen in the *Crim1*^*FLOX/FLOX*^*; WT1-CreERT2; R26R* and *Crim1*^*FLOX/FLOX*^*; WT1-Cre; R26R* hearts. This was observed alongside a reduction in the number of cardiac fibroblasts, one of the major derivatives of the epicardium^1^,^3^,^45^. Cardiac fibroblasts represent a crucial component of the myocardium, secreting a large number of ECM molecules required to support other resident cells of the heart, as well as growth factors and cytokines for the proper functioning of these cells^46^. However, there is little known about the factors that contribute to cardiac fibroblast fate specification. These EPDCs that have lost Crim1 could remain undifferentiated cells residing in the compact myocardium, or preferentially ‘switch’ to coronary vascular smooth muscle cells rather than cardiac fibroblasts. Cardiac fibroblasts generated after injury are crucial to cardiac repair in terms of both the ECM molecules secreted as well as overall cardiac performance^29^,^47^. Our data indicate a cell-autonomous requirement for Crim1 in the production of cardiac fibroblasts. Further studies are required to validate the role of Crim1 in lineage specification into fibroblasts and to also address whether it plays a cell-autonomous role in the differentiation of EPDCs into coronary vascular smooth muscle cells.

In summary, this study reveals a role for epicardial Crim1 in normal heart development. Although thought to be a largely quiescent lining surrounding the myocardium, there has been significant recent focus on ways to reactivate the adult epicardium in response to injury and disease^48^. This revolves around re-expression of embryonic epicardial genes and the release of paracrine and autocrine factors from the epicardium or EPDCs^48^. An active participant in epicardially-regulated heart development, Crim1 modulation may therefore provide a potential avenue for future molecular and cellular therapeutic interventions in cardiac regeneration and repair. Moreover, whether or not abnormal CRIM1 expression plays a role in congenital human heart disease will be an important topic for future investigations.

## Methods

### Ethics statement

The work performed in this study conformed to The University of Queensland’s Animal Welfare Unit guidelines for animal use in research, and followed the National Institutes of Health Guide for the Care and Use of Animals and the Australian Code of Practice for the Care and Use of Animals for Scientific Purposes. All experimental protocols were approved by The University of Queensland’s Animal Ethics Unit (Animal Ethics Committee approval numbers: AIBN/274/08/NHMRC, SMBS/420/11/NHMRC).

### Animal breeding

The mouse lines used in this study were the *Crim1*^*FLOX*^ conditional mutant^15^, *Crim1*^Δ*flox*^ knock-out^15^ and *Crim1*^*KST264*^ genetrap line^11^, *ROSA26 Cre*-reporter line (*R26R*)^49^, *WT1-Cre* line^25^, *WT1-CreERT2* line^24^,^50^ and *Mlc2v-Cre* line^20^. The *Crim1*^*KST264*^ mouse line was created as part of a genetrap screen^51^, with insertion of the β-Geo cassette into intron 1 of *Crim1* resulting in the fusion of exon 1 and the β-Geo cassette within the *Crim1* transcript, thereby creating a hypomorphic allele which expresses a minor, alternately spliced isoform of CRIM1^11^. The *Crim1*^*FLOX*^ conditional mutant mouse line was generated, using Cre/LoxP recombination, by flanking exons 3 and 4 with unidirectional LoxP sites^15^. These *Crim1*^*FLOX*^ mice were crossed with a line that expressed Cre recombinase ubiquitously (*CMV-Cre*) to produce the *Crim1*^Δ*flox*^ line. This deletion created an out-of-frame transcript and a predicted non-functional protein and as such, Crim1 is deleted from all tissues in these mice^15^. All lines were maintained on a C57Bl6 genetic background. Embryos were obtained from timed matings between *Crim1*^+*/*Δ*flox*^ intercrosses, *Crim1*^+*/KST264*^ intercrosses, or *Crim1*^*FLOX/FLOX*^*; R26R* females mated with *Crim1*^+*/FLOX*^; *WT1-Cre*, *Crim1*^+*/FLOX*^; +/*WT1-CreERT2* or *Crim1*^+*/FLOX*^; *Mlc2v-Cre* males.

### Sample preparation

Mouse embryos were obtained from timed matings between *Crim1*^+*/*Δ*flox*^ intercrosses or *Crim1*^+*/KST264*^ intercrosses, with the presence of a vaginal plug regarded as 0.5 days post coitum (dpc). Embryonic hearts were dissected in PBS and fixed for 2 hours in 4% PFA/PBS at 4 °C, washed in PBS, and photographed in whole-mount. Samples were then dehydrated and processed for paraffin infusion and embedding, and 7 μm sections were cut on a microtome. Hematoxylin and eosin staining was performed using standard protocols. Embryonic hearts collected from matings between *Crim1*^+*/*Δ*flox*^ intercrosses collected at E13.0 and E13.5 were also fixed in 4% PFA/PBS at 4 °C, washed in PBS, cryo-protected in 30% sucrose/PBS at 4 °C, embedded in OCT (Tissue-Tek) and 10 μm sections were cut on a cryostat and air-dried before proceeding with the immunofluorescence procedure.

Timed matings were set up between *Crim1*^+*/FLOX*^; *WT1-Cre* males and *Crim1*^*FLOX/FLOX*^*; R26R* females to produce *Crim1*^+*/FLOX*^; *WT1-Cre; R26R* and *Crim1*^*FLOX/FLOX*^; *WT1-Cre; R26R* embryos collected at 15.5 dpc (at this stage, the myocardium has expanded and cells derived from the epicardium have started to differentiate into various lineages), *Crim1*^+*/FLOX*^; +/*WT1-CreERT2* males and *Crim1*^*FLOX/FLOX*^*; R26R* females, for the production of *Crim1*^+*/FLOX*^; +/*WT1-CreERT2*; *R26R* and *Crim*^*FLOX/FLOX*^; +/*WT1-CreERT2; R26R* embryos collected at 15.5 dpc and 17.5 dpc (at 17.5 dpc, cells derived from the epicardium have differentiated into cardiac fibroblasts, coronary vascular smooth muscle cells, and a small proportion of coronary vascular endothelial cells), and between *Crim1*^+*/FLOX*^; *Mlc2v-Cre* males and *Crim1*^*FLOX/FLOX*^*; R26R* females to produce *Crim1*^+*/FLOX*^; *Mlc2v-Cre*; *R26R* and *Crim1*^*FLOX/FLOX*^; *Mlc2v-Cre*; *R26R* embryos collected at 13.5 dpc (the myocardium has begun to expand at this stage through proliferation of cardiomyocytes).

Embryonic samples from the *WT1-Cre, WT1-CreERT2* and *Mlc2v-Cre* (β-galactosidase activity being a readout of *Cre* activity based on the R26R reporter) and *Crim1*^+*/KST264*^ intercrosses (β-galactosidase activity being under the control of the endogenous Crim1 promoter) were X-Gal-stained in whole-mount as previously described^6^ before processing for paraffin embedding and sectioning as described above. Some X-Gal-stained sections were counterstained with nuclear fast red (NFR; Vector Laboratories). Others were subjected to cell biological and immunohistochemical analyses as detailed below.

### Tamoxifen administration

Tamoxifen (MP Biomedicals, 02156738) was dissolved in corn oil at a concentration of 20 mg/ml. To induce *WT1-CreERT2*^24^,^50^, two doses of 2 mg tamoxifen^52^ were injected intraperitoneally to pregnant dams (at 9.5 dpc and 10.5 dpc), prior to harvesting the mouse embryos.

### Scanning electron microscopy

13.5 dpc embryos were collected from intercrosses of *Crim1*^+*/*Δ*flox*^ mice, and the hearts were dissected and fixed in 2.5% glutaraldehyde in PBS for 1 hour at room temperature. Samples were then washed 3 times with PBS and stored at 4 °C until processing for scanning electron microscopy as previously described^53^. Scanning electron microscopy was performed using a CM-500 Benchtop Scanning Electron Neoscope.

### Antibodies

Primary antibodies/lectin used in the study were: rabbit polyclonal anti-phospho-Histone H3 (pSer10; Millipore #06-570); rabbit monoclonal anti-cleaved caspase 3 (activated caspase 3; Cell Signalling Technology #9664); rabbit monoclonal anti-phospho-AKT (pSer473; D9E; Cell Signalling Technology #4060); rabbit monoclonal anti-phospho-ERK1/2 (pThr202/pTyr204; D13.14.4E; Cell Signalling Technology #4370); rabbit monoclonal anti-phospho-SMAD1/5 (pSer463/pSer465; Invitrogen #700047); rabbit polyclonal anti-phospho-SMAD2 (pSer465/pSer467; Millipore AB3849); mouse monoclonal anti-smooth muscle α-actin (clone 1A4, Santa Cruz Biotechnology, sc-3225); rabbit anti-β-catenin (whole antiserum; Sigma Aldrich #C2206); rabbit polyclonal SM22 alpha (Abcam Ab14106); rabbit polyclonal anti-periostin (Abcam Ab92460); mouse monoclonal anti-Wilms’ Tumor 1 (WT1; Dako #M3561); MF20 (supernatant, Developmental Studies Hybridoma Bank) and Isolectin B4 (Invitrogen). Secondary antibodies for immunofluorescence experiments were Alexa Fluor-488, −594 or −633-conjugated secondary antibodies (Invitrogen). TSA plus Cyanine 3 system was used for signal amplification in the case of pERK1/2 and pAKT (PerkinElmer #NEL744001KT) Secondary antibodies for immunohistochemistry experiments were anti-rabbit HRP (Promega; #W4011), anti-mouse HRP (Promega; #W4021), anti-rabbit biotin (Sigma; # B7389). The Vectastain Elite ABC kit (Standard) was also used (Vector Laboratories; #PK6100).

### Immunohistochemistry

Experiments using mouse antibodies were blocked with mouse-on-mouse (Vector MOM kit; BMK 2202) blocking reagent for one hour. Sections were primed with MOM diluent for five minutes after washes in 1 × PBS twice. Mouse primary and secondary antibodies were diluted in MOM protein diluent. Immunohistochemistry using pHH3 was performed and sections counterstained with 1% DAB followed by NFR staining. Immunohistochemistry to detect periostin used anti-rabbit biotin and Vectastain ABC as secondary and tertiary antibodies. Vectastain ABC reagents were prepared as per manufacturer’s protocols. After one-hour incubation with anti-rabbit biotin, Vectastain ABC was added for 30 minutes in room temperature, followed by hematoxylin staining. Indirect immunofluorescence on tissue sections was performed on paraformaldehyde-fixed, paraffin-embedded samples as described^6^. For immunofluorescence involving detection of cleaved caspase 3 or PHH3, paraffin sections were subject to heat-induced antigen retrieval in a citrate buffer (pH 6.0). Immunofluorescence on cryosectioned samples was performed to detect β-Catenin, MF20, phospho-SMAD1/5, phospho-SMAD2, phospho-AKT and phospho-ERK1/2. Tyramide amplification according to manufacturer’s methods was used for signal amplification of phospho-ERK1/2 and phospho-AKT.

### Primary epicardial explant cultures and migration assays

11.5 dpc embryos were collected from intercrosses of *Crim1*^+*/KST264*^ mice, and the ventricular component was dissected from the rest of the heart in sterile PBS and cultured in DMEM supplemented with 10% FCS and penicillin/streptomycin in 4-well tissue culture plates. Once the epicardium had grown out from the primary culture, the ventricular mass was removed. The epicardial cells were grown to confluence and were passaged once and plated at a high confluence. After two days of further culture, the primary epicardial cells were used in a modified Boyden chamber assay. Epicardial cultures were collected after trypsin treatment to completely dissociate cells, washed and resuspended in DMEM (with 10% FCS). 1,000 cells in identical volumes were placed in the upper chamber of 6.5 mm transwell culture inserts (polycarbonate filter, 5.0 μm pore size, Corning). After 16 hours of culture, the cells in the upper part of the transwell inserts were removed using a cotton swab, and the cells that had migrated through the polycarbonate filter were quantified after fixation in 4% PFA in PBS and staining with DAPI. Epicardial identity was confirmed after enrichment by immunofluorescence for the transcription factor WT-1^24^,^54^, using standard techniques as described^6^.

### In situ hybridization

Section *in situ* hybridization was performed as previously described^55^. Whole-mount *in situ* hybridization was performed as previously described^56^ with minor changes. Hybridization was performed with a riboprobe concentration of 0.4 μg/mL in pre-hybridization solution. Color detection was performed with the chromogenic substrate NBT/BCIP (Roche). Samples were then washed, post-fixed in 4% PFA/PBS, and photographed. At least three embryos of each genotype/gene probe were analyzed. Some whole-mount samples were processed for paraffin infusion and sectioned as described above.

### Quantitative real-time PCR analysis

Ventricular samples from *Crim1*^+*/*+^ and *Crim1*^Δ*flox/*Δ*flox*^ hearts were microdissected, homogenized and total RNA was extracted using an RNeasy Mini Kit (Qiagen, #74104). Reverse transcription was performed using SuperscriptIII (Invitrogen) and qPCR was performed. Briefly, 500 ng total RNA was reverse-transcribed with random primers and dNTPs. cDNA was diluted 1/5 with RNase/DNase-free water. qPCRs were carried out in a QuantStudio6 (Applied Biosystems) using SYBR green (Takara) and standard qPCR conditions. The data were analyzed with the QuantStudio6 software, with TFIID used as a relative standard. All samples were tested in triplicate. Relative transcript levels were assessed using the ΔCt method. Statistical analyses were performed using a two-tailed unpaired t-test. The primer sequences used in this study were purchased from Sigma-Aldrich, and are detailed below:

*Periostin:* Forward 5′-AAGCTGCGGCAAGACAAG-3′

*Periostin:* Reverse 5′-TCAAATCTGCAGCTTCAAGG-3′

*Collagen1a:* Forward 5′-AGACATGTTCAGCTTTGTGGAC-3′

*Collagen1a:* Reverse 5′-GCAGCTGACTTCAGGGATG-3′

*Collagen3a:* Forward 5′-ACGTAGATGAATTGGGATGCAG-3′

*Collagen3a*: Reverse 5′-GGGTTGGGGCAGTCTAGTG-3′

*Collagen5a:* Forward 5′-CTACATCCGTGCCCTGGT-3′

*Collagen5a:* Reverse 5′-CCAGCACCGTCTTCTGGTAG-3′

*Transgelin:* Forward 5′-CCTTCCAGTCCACAAACGAC-3′

*Transgelin*: Reverse 5′-CTAGGATGGACCCTTGTTGG-3′

*Alpha smooth muscle actin:* Forward 5′-ACTCTCTTCCAGCCATCTTTCA-3′

*Alpha smooth muscle actin:* Reverse 5′-ATAGGTGGTTTCGTGGATGC-3′

*Myosin heavy chain 11:* Forward 5′-ACGCCCTCAAGAGCAAACT-3′

*Myosin heavy chain 11:* Reverse 5′-CCCTTCTGGAAGGAACAATG-3′

*Calponin1:* Forward 5′-GAAGGTCAATGAGTCAACTCAGAA-3′

*Calponin1:* Reverse 5′-CCATACTTGGTAATGGCTTTGA-3′

*Myosin heavy chain 7:* Forward 5′-TGCTGAGGCCCAGAAACAAGTG-3′

*Myosin heavy chain 7:* Reverse 5′-CTGGATTTGAGTGTCCTTCAGCAG-3′

*Nkx2-5:* Forward 5′-ATTGACGTAGCCTGGTGTCTCG-3′

*Nkx2-5:* Reverse 5′-AGTGTGGAATCCGTCGAAAGTGC-3′

*Tie2:* Forward 5′-AGAGGCCGAACATTCCAAGTAGC-3′

*Tie2:* Reverse 5′-GGCCAAACAAACTGGCTTCTGC-3′

*PDGFRα:* Forward 5′-TTGCACAGCCTCTGTTTGTTGC-3′

*PDGFRα:* Reverse 5′-ATGGTGTCATGCAAGGCCCAAAG-3′

*PDGFRβ:* Forward 5′-CAGTCCCGGCTACCCTATCT-3′

*PDGFRβ:* Reverse 5′-GACCCAGAGTTTTCTCGGCA-3′

*Tcf21:* Forward 5′-CATTCACCCAGTCAACCTGA-3′

*Tcf21:* Reverse 5′-CCACTTCCTTCAGGTCATTCTC-3′

*TFIID:* Forward 5′-ACGGACAACTGCGTTGATTTT-3′

*TFIID:* Reverse 5′-ACTTAGCTGGGAAGCCCAAC-3′

### Imaging and data analysis

Bright-field images of tissue sections were captured using an Olympus BX-51 BF/DF slide microscope with Canon digital cameras with DP Controller software (Olympus). Whole-mount images were captured using either an Olympus SZX-12 stereo-microscope with DP Controller software, or a Zeiss Stemi 2000 stereo-microscope with an AxioCam digital camera with AxioVision software (Zeiss). Fluorescent images of tissue sections were captured on an Olympus IX-81 laser scanning confocal microscope and an Olympus FV1000 upright laser scanning confocal microscope with Olympus Fluoview software (Ver3.1a). Images were adjusted for colour levels, brightness and contrast, and figures compiled, using Adobe Photoshop software. The number of samples included in data averages is indicated in the figure legends where applicable. To determine the statistical significance of the prevalence of phenotypes among mutant and control groups, a Z-test was used (Fig. 2N). Epicardial defects examined and quantified include either a loss of squamous morphology or blebbing. For the analysis of 13.0 dpc compact myocardium thickness and cell density (Fig. 3), sections were immunolabelled for Acta2 and PECAM-1, and nuclei counter-stained with DAPI as described above. 40× fields of view of the ventricular compact myocardium were imaged on a laser scanning confocal microscope on each of three–five non-consecutive sections for each sample analyzed. The compact myocardium was considered as the actin-positive area of the ventricular chambers, bounded on the endocardial side by the base of the trabeculae (delineated by PECAM-1 staining). To determine cell density, the area was determined from confocal images using either Olympus Fluoview or ImageJ software, and the number of DAPI-stained nuclei quantified using ImageJ software. For the quantification of the pHH3-positive and CC3-positive cells at 13.0 dpc, immunolabelling was performed as described above. The ventricular compact myocardium was imaged on a laser scanning confocal microscope (multiple images at 40× objective) on five–seven non-consecutive sections for each sample analyzed. Cells were scored as epicardial or sub-epicardial if they were on the periphery of the section and were actin-negative. Statistical significance between pooled data from mutant and control groups was determined using a two-tailed, unpaired Student’s t-test.

For the analysis of epicardium-derived cells using the using the *WT1-CreERT2* line, 2–4 non-consecutive, coronal sections from each sample (X-Gal-positive; *WT1-CreERT2*) (Fig. 4I,M) were quantified and the average of X-Gal-positive cells in the mid-ventricular region per sample was measured. For analysis using the *WT1-Cre line*, 16 non-consecutive, coronal sections from each sample (X-Gal-positive; *WT1-Cre*) were quantified and the total number X-Gal-positive cells in the ventricles was counted (data not shown). Cells were scored as epicardial, sub-epicardial if they were on the periphery of the section, or intramyocardial. For the analysis of the location of epicardium-derived cells (X-Gal-positive, *WT2-CreERT2/WT1-Cre*; epicardial, sub-epicardial, or intramyocardial), a two-way ANOVA with Bonferroni’s post-test was used.

For quantification of β-Catenin (Fig. 5Q–U), sections were co-immunolabelled for MF20 and 40× images were acquired using a laser scanning confocal microscope from at least 2 sections for each sample. Cells were scored as epicardial or sub-epicardial if they were on the periphery of the section and were MF20-negative. β-Catenin accumulation at adherens junctions between epicardial cells^27^ was quantified, with the junctions being defined as points of contact between two epicardial cells. Statistical significance between pooled data from mutant and control groups was determined using a two-tailed, unpaired Student’s t-test.

For quantification of cardiac fibroblasts and proliferating EPDCs (Fig. 6), 20X images were acquired using a slide microscope from a minimum of three non-consecutive sections for each sample. The number of X-Gal- and periostin-positive and X-Gal- and pHH3-positive cells were counted using ImageJ software and data analyzed using two-tailed, unpaired Student’s t-test and two-way ANOVA respectively. Positive cells were identified as those that possessed both blue β-gal and a corresponding brown nucleus denoted by DAB staining.

For quantification of phospho-SMAD levels (Fig. 5A–H, Supplementary Fig. 6A,H), 40× images were acquired with a laser scanning confocal microscope from at least five sections for each sample and for phospho-ERK1/2 and phospho-AKT levels (Fig. 5I–P, Supplementary Fig. 6I–P), from at least 2 sections for each sample. To determine average signal intensity/cell, unsaturated confocal images were used, and signal intensity determined using the Integration function in Olympus Fluoview software. The number of DAPI-stained nuclei in the demarcated area was quantified using ImageJ software. Average signal per cell determined was divided by 1000 for graphical representation. Statistical significance between pooled data from mutant and control groups was determined using a two-tailed, unpaired Student’s t-test. Quantified data shown are mean ± standard deviation.

## Additional Information

**How to cite this article**: Iyer, S. *et al.* Crim1 has cell-autonomous and paracrine roles during embryonic heart development. *Sci. Rep.* **6**, 19832; doi: 10.1038/srep19832 (2016).

## Supplementary Material

Supplementary Information
