# Supplementary material for: Crim1 has cell-autonomous and paracrine roles during embryonic heart development
Source: Sci Rep. 2016 Jan 29;6:19832. doi: 10.1038/srep19832 (PMC4731764; doi:10.1038/srep19832)
Supplement: Supplementary Information [file srep19832-s1.pdf]

Supplementary Information

Crim1 has cell-autonomous and paracrine roles during embryonic heart development.

Swati Iyer[1], Fang Yu Chou[1], Richard Wang[1], Han Sheng Chiu[1], Vinay K. S. Raju[1], Melissa H. Little[2,3,4], Walter G. Thomas[1], Michael Piper[1,5#], David J. Pennisi[1#].

[1]School of Biomedical Sciences, The University of Queensland, Brisbane, 4072, Australia, [2]Institute for Molecular Bioscience, The University of Queensland, Brisbane, 4072, Australia, [3]Murdoch Children's Research Institute, Melbourne, [4]Department of Pediatrics, University of Melbourne and [5]Queensland Brain Institute, The University of Queensland, Brisbane, 4072, Australia.

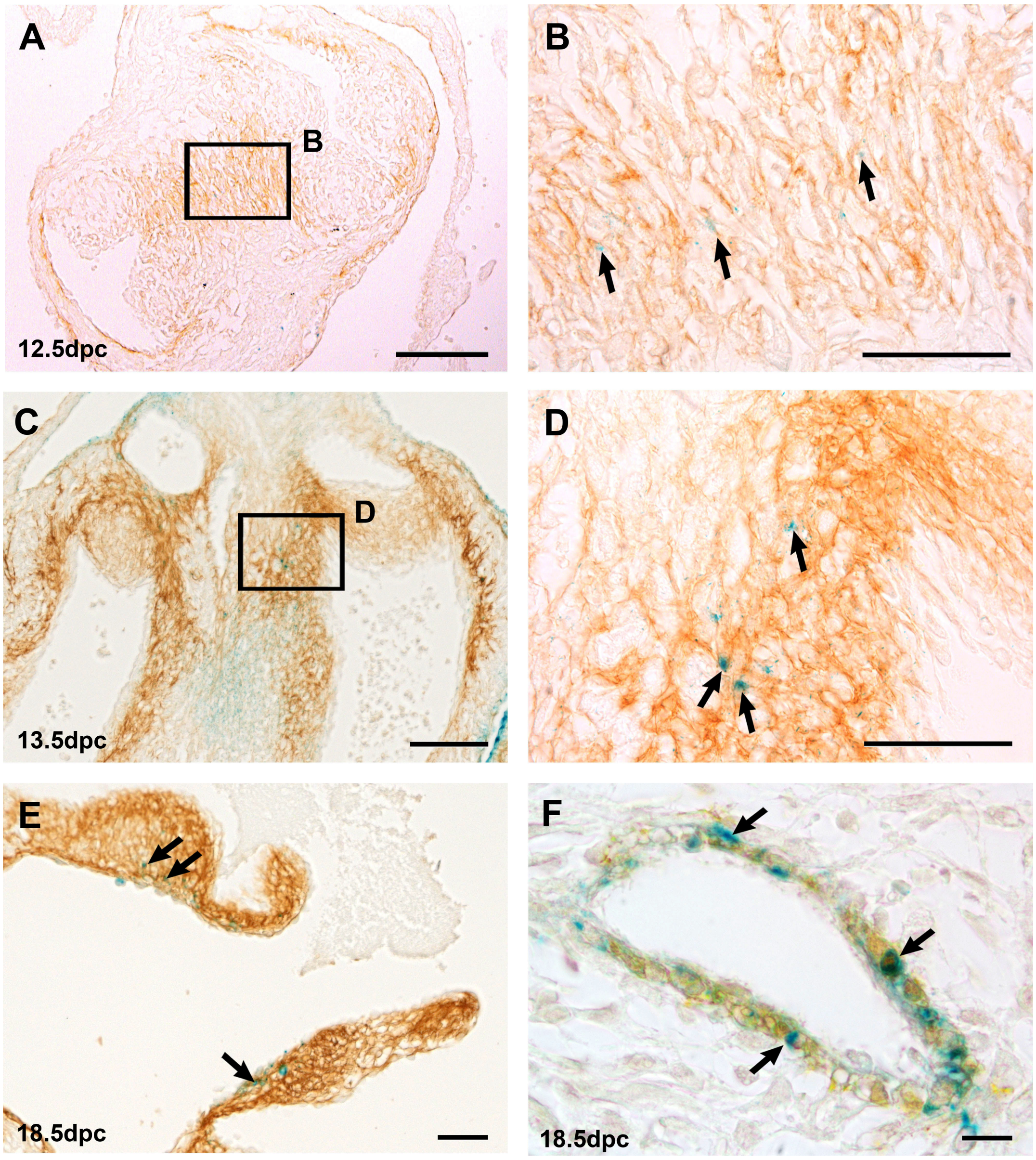

Supplementary figure 1. Crim1 is expressed in the outflow tract, mitral valve leaflets and coronary vascular smooth muscle cells.

A, Periostin immunohistochemistry on an X-Gal stained *Crim1*$^{+/KST264}$ transverse section at 12.5 dpc. B, higher magnification view of the boxed area in A, showing periostin- and X-Gal-positive outflow tract mesenchymal cells (arrows). C, Periostin immunohistochemistry on an X-Gal stained *Crim1*$^{+/KST264}$ coronal section at 13.5 dpc. D, higher magnification view of boxed area in C, showing periostin- and X-Gal-positive mesenchymal cells (arrows). E,

Periostin immunohistochemistry on an X-Gal stained *Crim1*$^{+/KST264}$ coronal section at 18.5 dpc, showing periostin- and X-Gal-positive mitral valve leaflet cells (arrows). F, Transgelin immunohistochemistry on an X-Gal stained *Crim1*$^{+/KST264}$ coronal section at 18.5 dpc, showing transgelin- and X-Gal-positive coronary vascular smooth muscle cells (arrows). Scale bars, A, C, 100 µm; B, D, E, 40 µm; F, 10 µm.

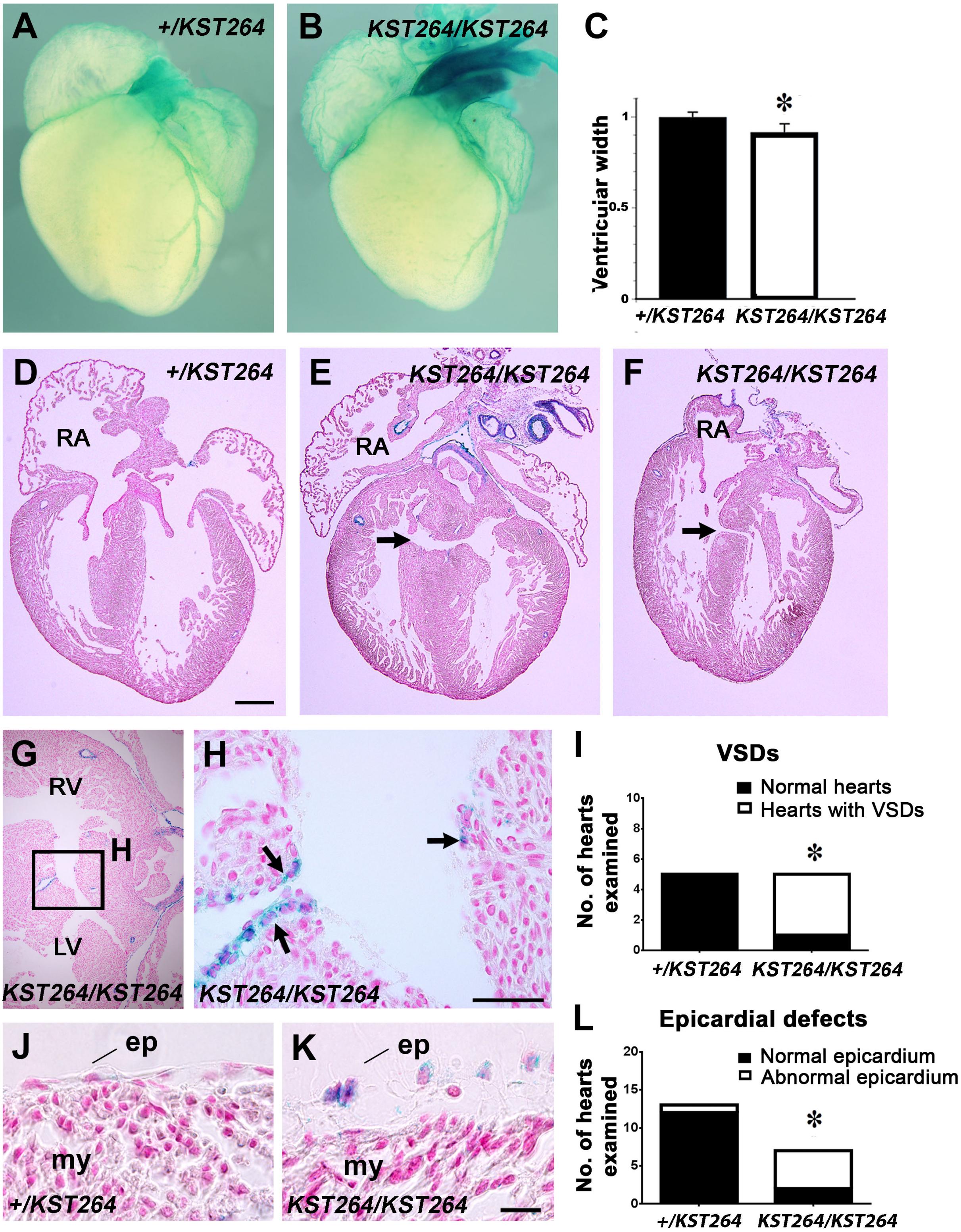

Supplementary figure 2. Congenital heart defects in *Crim1*$^{KST264/KST264}$ embryos.

A–C, 18.5 dpc littermate hearts stained with X-Gal to reveal Crim1-LacZ expression (blue). A, *Crim1*$^{+/KST264}$ 18.5 dpc heart. B, *Crim1*$^{KST264/KST264}$ 18.5 dpc heart. C, Quantification of the

size of the ventricular component at 18.5 dpc, showing a significant reduction in the width of *Crim1*$^{KST264/KST264}$ hearts (n=7–9). D-F, Coronal sections of 18.5 dpc hearts stained with X-Gal and counter-stained with nuclear fast red. The right atrium (RA) is labelled. D, *Crim1*$^{+/KST264}$ 18.5 dpc heart showing normal morphology. E,F, *Crim1*$^{KST264/KST264}$ hearts displaying a prominent VSD, a communication between left and right ventricles (arrow). G, Another section of the heart in F, showing the VSD. H, View of the inset in G. Note the Crim1-LacZ-positive cells (arrows) in the IVS either side of the communication between the ventricles. I, Quantification of prevalence of VSDs in *Crim1*$^{+/KST264}$ and *Crim1*$^{KST264/KST264}$ hearts (n=5). Z-Score -2.582. *, P<0.01, J-L, Differing ventricular epicardial morphology between *Crim1*$^{+/KST264}$ and *Crim1*$^{KST264/KST264}$ hearts. The epicardium (ep) appears less squamous-like in *Crim1*$^{KST264/KST264}$ hearts (my, myocardium). L, Quantification of prevalence of epicardial defects, (n=13-7). Z-Score -2.7118. *, P<0.01. LV, left ventricle; RV, right ventricle. Scale bars D, 500 µm; H, 50 µm; K, 10 µm.

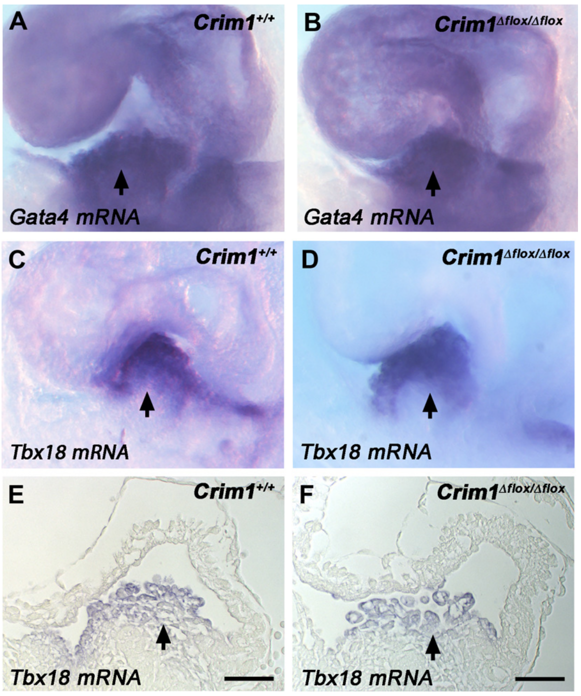

Supplementary figure 3. Crim1 is not necessary for specification or early development of the proepicardium.

A-F, Micrographs of 9.5 dpc embryos from *Crim1*$^{+/+}$ (A, C, E) and *Crim1*$^{\Delta flox/\Delta flox}$ (B, D, F) embryos stained for *Gata4* mRNA expression (purple stain, A and B) and *Tbx18* mRNA expression (purple stain, C and D). E, F, Micrographs of histological sections of whole-mount *in situ* hybridization-stained embryos showing *Tbx18* expression. The PE is indicated (arrows). Scale bars A and E, 50 µm.

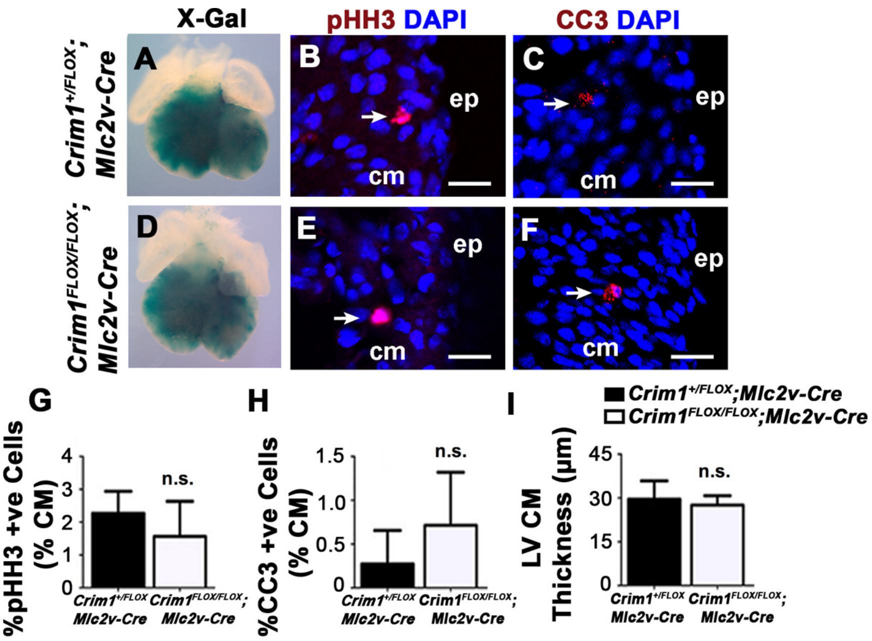

Supplementary figure 4. Myocardial deletion of Crim1 does not affect cardiomyocyte proliferation and survival and thickness of compact myocardium.

A, D representative whole mount images of X-Gal stained *Crim1*$^{+/FLOX}$; *Mlc2v- Cre; R26R* and *Crim1*$^{FLOX/FLOX}$*; Mlc2v-Cre; R26R* hearts at 13.5 dpc. B, E merged confocal images of DAPI (blue) and pHH3 (red), with a pHH3-positive nucleus (arrow). C, F merged confocal images of DAPI (blue) and CC3 (red), with a CC3-positive nucleus (arrow). G, No significant difference in percentage of pHH3-positive cells in the compact myocardium of left and right ventricles in control and mutant hearts (n=4-6). H, No significant difference in percentage of CC3-positive cells in the compact myocardium of left and right ventricles in control and mutant hearts (n=4-5). I, No significant difference in thickness of compact myocardium in the left ventricle of control and mutant hearts (n=3). cm, compact myocardium; ep, epicardium; n.s, not significant. Scale bars B, C, E, F 20 µm.

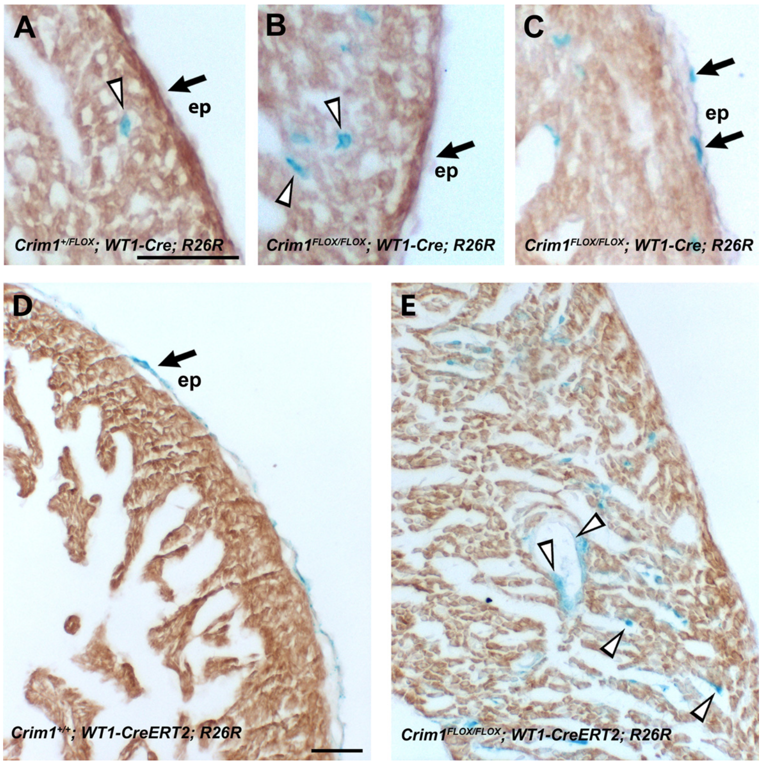

Supplementary figure 5. Fidelity of the *WT1-Cre* and *WT1-CreERT2* lines.

A-E, MF20 immunohistochemistry performed on A-C, 15.5 dpc *WT1-Cre* X-Gal stained sections showing MF20-negative epicardium (arrows) and MF20-negative EPDCs (open arrowheads), D, *WT1-CreERT2* 13.5 dpc X-gal stained section showing that most epicardial cells are X-Gal-positive (arrow), and E, *WT1-CreERT2* 17.5 dpc X-Gal stained section showing MF20-negative EPDCs (open arrowheads), confirming that the EPDCs are not cardiomyocytes. ep; epicardium. Scale bars A-C, 25μm; D-E, 50 μm.

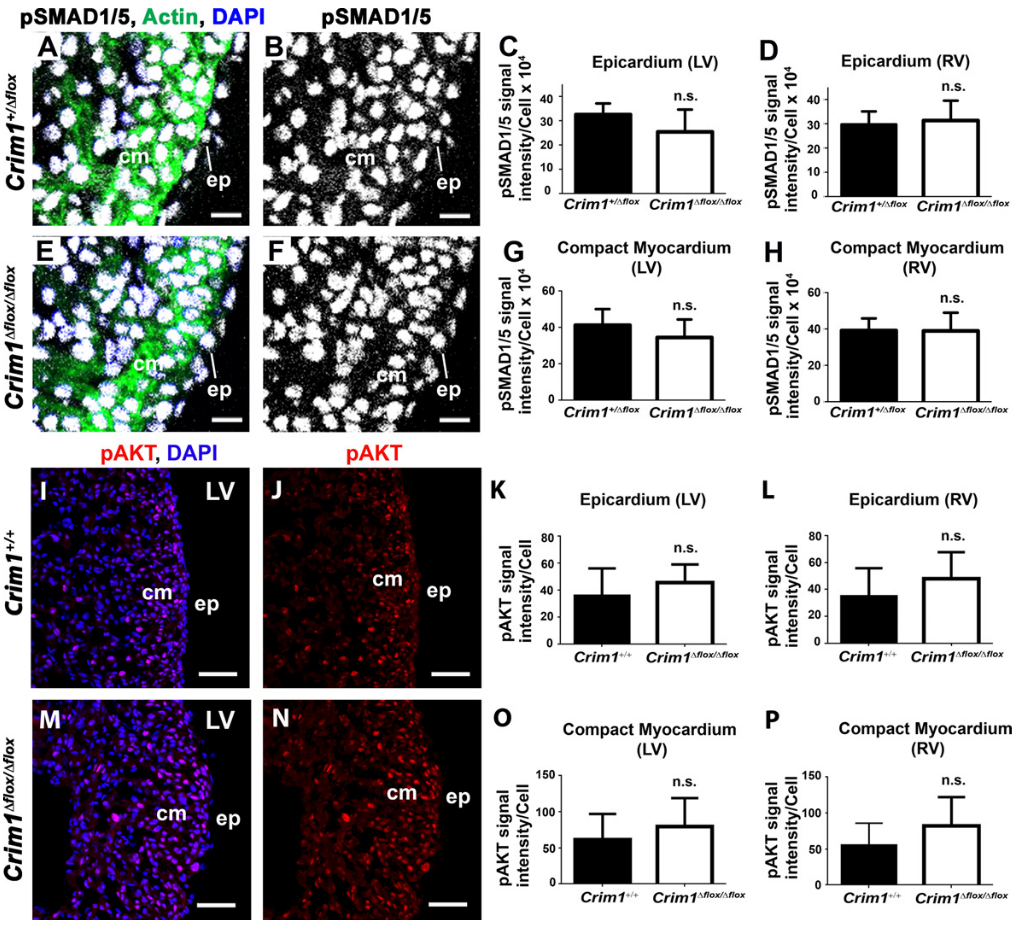

Supplementary figure 6. Phospho-SMAD1/5 and phospho-AKT levels are not significantly changed in the epicardium or myocardium of $^{\mathrm{Crim1}\Delta\mathrm{flox}/\Delta\mathrm{flox}}$ hearts.

Confocal images of ventricular sections of 13.0 dpc hearts from *Crim1*$^{+/\Delta flox}$ (A, B) and *Crim1*$^{\Delta flox/\Delta flox}$ (E, F) embryos stained for phospho-SMAD1/5 (white). A and E, merged images of DAPI (blue) and actin (green) to delineate the myocardium with the phospho-SMAD1/5 (white) from A and E, respectively. C, D, G, and H, Quantification of the average signal intensity/cell after indirect immunofluorescence to detect phospho-SMAD1/5 showed there was no change in the levels in the epicardium or compact myocardium of left and right ventricles of *Crim1*$^{\Delta flox/\Delta flox}$ hearts in comparison to controls (n=5). Confocal images of ventricular sections of 13.5 dpc hearts from *Crim1*$^{+/+}$ (I, J) and *Crim1*$^{\Delta flox/\Delta flox}$ (M, N) embryos stained for phospho-AKT (red). Quantification of signal intensity/ cell after

immunofluorescence to detect phospho-AKT revealed no change in the levels in the epicardium or compact myocardium of left and right ventricles of *Crim1*$^{\Delta flox/\Delta flox}$ hearts in comparison to controls (n=5-6). cm, compact myocardium; ep, epicardium; n.s., not significant. Scale bars, A, B, E, F 10µm; I, J, M, N, 50 µm.

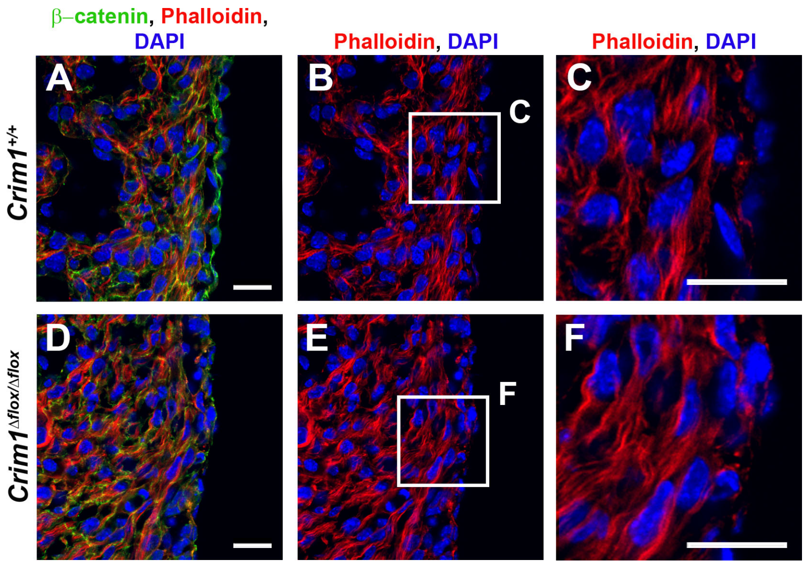

Supplementary figure 7. No visible change in filamentous actin morphology in the epicardium or myocardium of *Crim1*$^{\Delta flox/\Delta flox}$ hearts at 13.5 dpc.

A-F, Representative confocal images of ventricular sections of 13.5 dpc hearts from *Crim1*$^{+/+}$ (A, B, C) and *Crim1*$^{\Delta flox/\Delta flox}$ (D, E, F) hearts at 13.5 dpc. A, D merged images of DAPI (blue) and MF20 (green) to delineate the myocardium with Phalloidin (red) from B and E, respectively (n=4-5). C, F, magnified views of boxed regions in B and E respectively. Scale bars A – F, 20 µm.

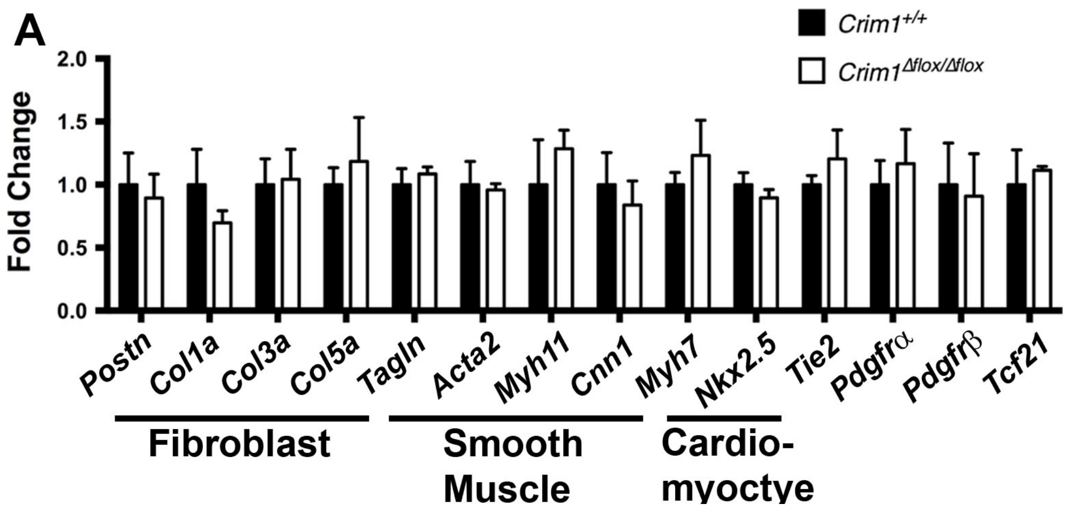

Supplementary figure 8. No change in the expression of key cardiac genes in *Crim1*$^{\Delta flox/\Delta flox}$ hearts at 17.5 dpc.

A, qPCR with fibroblast, smooth muscle and cardiomyocyte markers showing no significant difference in fold change between *Crim1*$^{+/+}$ and *Crim1*$^{\Delta flox/\Delta flox}$ ventricular samples at 17.5 dpc. Note that for *Collagen1a*, P=0.0556. (n=4-5).
